# Multi-Channel Convolutional Neural Network Based 3D Object Detection for Indoor Robot Environmental Perception

**DOI:** 10.3390/s19040893

**Published:** 2019-02-21

**Authors:** Li Wang, Ruifeng Li, Hezi Shi, Jingwen Sun, Lijun Zhao, Hock Soon Seah, Chee Kwang Quah, Budianto Tandianus

**Affiliations:** 1 State Key Laboratory of Robotics and System, Harbin Institute of Technology, Harbin 150001, China; 15b908017@hit.edu.cn (L.W.); 18S008061@stu.hit.edu.cn (J.S.); 2 EON Reality Pte Ltd, Singapore 138567, Singapore; hezi.shi@eonreality.com; 3 School of Computer Science and Engineering, Nanyang Technological University, Singapore 639798, Singapore; ashsseah@ntu.edu.sg; 4 ST Electronics (Training & Simulation Systems) Pte Ltd, Singapore 567714, Singapore; quah.cheekwang@stee.stengg.com; 5 ST Engineering-NTU Corporate Laboratory, School of Electrical and Electronic Engineering, Nanyang Technological University, Singapore 637335, Singapore; btandianus@ntu.edu.sg

**Keywords:** 3D object detection, multi-channel CNN, indoor robot, environmental perception

## Abstract

Environmental perception is a vital feature for service robots when working in an indoor environment for a long time. The general 3D reconstruction is a low-level geometric information description that cannot convey semantics. In contrast, higher level perception similar to humans requires more abstract concepts, such as objects and scenes. Moreover, the 2D object detection based on images always fails to provide the actual position and size of an object, which is quite important for a robot’s operation. In this paper, we focus on the 3D object detection to regress the object’s category, 3D size, and spatial position through a convolutional neural network (CNN). We propose a multi-channel CNN for 3D object detection, which fuses three input channels including RGB, depth, and bird’s eye view (BEV) images. We also propose a method to generate 3D proposals based on 2D ones in the RGB image and semantic prior. Training and test are conducted on the modified NYU V2 dataset and SUN RGB-D dataset in order to verify the effectiveness of the algorithm. We also carry out the actual experiments in a service robot to utilize the proposed 3D object detection method to enhance the environmental perception of the robot.

## 1. Introduction

The traditional environmental perception of indoor service robots mainly solves the problems of localization, navigation, and obstacle avoidance in order to carry out autonomous movement. However, most of these studies focus on the description of the geometric information of an environment. The high-level perception of the environment for the indoor service robots requires more abstract information, such as semantic information like objects and scenes. With the rapid development of computer vision and artificial intelligence, many intelligent detection technologies have emerged, such as image-based object detection and scene recognition. If the service robot can recognize the common objects in the environment, it will greatly improve the environmental perception ability and the robustness of the robot, and provide a better way for human-robot interaction. However, image-based 2D object detection cannot provide the spatial position and size information of an object. Thus, it cannot satisfy the requirement of a service robot to operate in an indoor environment. Therefore, it is necessary to develop a 3D object detection technique in an indoor environment and to simultaneously recognize the object’s class, spatial position, and 3D size.

In recent years, research on convolutional neural networks has been achieved remarkable progress in the field of visual recognition. In the ImageNet Challenge, there are a large amount of emerging algorithms for image classification, such as AlexNet [1], GoogLeNet [2], and ResNet [3]. Subsequently, the R-CNN [4] algorithm is the first to apply the convolutional neural network algorithm to the object detection field in an image. Later, Fast R-CNN [5] and Faster R-CNN [6] have greatly improved the precision and efficiency of the object detection. In these algorithms, a feature map is obtained through convolutional neural network and spatial pyramid pooling [7] is employed to generate the fixed dimension vector of the proposal region in the feature map. Then, the classification and object position regression are realized by the classifier and the regressor.

2D object detection algorithms based on region proposals have matured recently. In the aspect of 3D object detection, 3D vehicle and pedestrian detection in the autonomous driving field have been extensively studied. An unmanned vehicle collects the outdoor environmental information through various sensors (3D laser, cameras, and so on) and recognizes vehicles, and pedestrians. The 3D bounding box is utilized to express the precise spatial position, direction, and 3D size. On the open dataset KITTI’s website [8,9] for autonomous driving, there are already hundreds of algorithms that have achieved good results in terms of detection accuracy and efficiency, such as the F-PointNet [10] algorithm, which can detect vehicles with a precision of 90% at moderate difficulty and a detection time of 0.17 s. F-PointNet [10] and VoxelNet [11] directly process input point cloud data through a convolutional neural network, which solves the problem of encoding and the feature extraction of disordered point clouds and obtains the end-to-end regression of a 3D bounding box. 3DVP [12], Mono3D [13], and 3DOP [14] extract only 3D proposal frames directly from a monocular image and estimate a 3D bounding box. MV3D [15], AVOD [16], and some other algorithms combine a laser point cloud with visual information and project the point cloud to a bird’s eye view (BEV) image. The information is entered into the convolutional neural network and multiple information is fused together to estimate a 3D bounding box.

However, there are fewer studies on 3D object detection for indoor environments compared to the autonomous driving field and a mature algorithm framework has not yet been formed. The dataset for indoor 3D object detection mainly includes NYU V2 [17] and SUN RGB-D [18] datasets, which consist of some common indoor objects, such as tables, chairs, beds, and so on, with the labeled 3D bounding boxes for objects. In an indoor environment, image and depth information are usually acquired by using RGB-D sensors such as Kinect. Currently, most algorithms employ RGB images or point clouds for object detection [19,20]. Since the BEV image provides information perpendicular to the camera viewpoint, the spatial distribution of the object can be clearly expressed. According to the authors’ knowledge, there is no such a method that utilizes the BEV image as the input information among the current indoor object 3D detection methods. Therefore, this paper proposes a multi-channel neural network system that combines the RGB, depth, and BEV images to achieve the 3D indoor object detection.

The main contributions of this paper are as follows: a multi-channel convolutional neural network-based 3D object detection for indoor robot environmental perception, which combines RGB, depth, and BEV imagesas the input. The BEV image is generated using point cloud projection and it is used as an input to the neural network to enhance the object detection accuracy. To the best of our knowledge, this is the first algorithm that combines the BEV image for 3D indoor object detection. In addition, a 3D proposal generation algorithm based on image semantics is proposed. Based on 2D image proposals, the category with the maximum probability is determined using statistical pixel semantic information, and the 3D proposal bounding box is determined according to the a priori size of the object.

The structure of this paper is organized as follows: Section 2 summarizes the recent research in the area of 3D object detection; Section 3 describes the proposed multi-channel CNN for 3D object detection in detail; Section 4 presents experimental results of the algorithm; and finally, Section 5 summarizes the content of the article.

## 2. Related Work

Object detection is one of the main tasks in both computer vision and robotics areas. Instead of recognizing and classifying objects, object detection requires placing a cuboid bounding box around the object. In the 2D object detection research area, there are some popular methods, such as selective search, which is based on a region proposal and DPM [21] (deformable parts model). Recently, development of the deep learning methods, such as RCNN [4], Fast-RCNN [5], Faster-RCNN [6], YOLO [22,23,24] and R-FCN [25], have highly improved the accuracy and efficiency of the 2D object detection task. However, the application of 2D object detection is still quite limited in robotics due to the lack of 3D information such as location, direction, and size. For example, if an indoor service robot recognizes a cup in the table from an RGB image, it does not know how to grasp it as there is no relative location of the cup in the space. With the emergence of 3D sensors, there have been numerous works that utilize 3D information to conduct 3D object detection such as obtaining the category, 3D size, and position of an object. Here, we briefly summarize some of the methods that utilize the depth information gained from the sensors to detect 3D objects from the environment.

### 2.1. Voxel and Point Cloud-Based Approach

Some of the existing works, such as Vote3Deep [26] and Deep Sliding Shapes [27], represent the 3D point clouds with the voxel and extend the general image CNN methods to 3D space. However, these methods are limited because of disparity of the point clouds and the high cost of computation. Surprisingly, PointNet [28], PointNet++ [29], and Frustum PointNets (F-PointNet) [10] provide a method that can directly cope with the raw unordered point clouds. As the F-PointNet method employs 2D object detection from images, the missed 2D detections will also lead to missed 3D detections.

### 2.2. 3D Multi-View Approach

In order to extract better and useful information from point clouds, and to improve the efficiency of detection, the multi-view work [30] represents the volumetric information of point cloud from different views. It provides an evaluation of how views affect the detection accuracy and present a strong multi-view (MV) classifier that accounts for different object views. MV3D [15] combines the region proposal method with a multi-view representation. The network extracts 3D proposals from the bird’s eye view (BEV) and then 3D proposals are projected to the BEV, front view, and RGB image to obtain 2D proposals. AVOD [16] also utilizes RGB and BEV images to extract features and fuses them to get 3D proposals for regression. A multi-view approach is always used in the autonomous driving area as the bird view image preserves the physical information of objects and avoids potential occlusion. We find that this method can also be used for indoor scene object detection as the BEV image can also describe the different information of objects compared to merely using RGB and depth images. In this paper, we employ the BEV to enhance the 3D object detection in an indoor environment.

### 2.3. 2.5D Image Approach

Another way to achieve the goal is to treat a depth image the same as a color image and extract features. The previous work [31] fuses the information from color images and depth images at an early stage and trains pose-based classifiers for 2D pedestrian detection. The paper [32] also includes a multimodality component in their framework that explores the fusion of RGB and depth images obtained by high-definition light detection and ranging. The other paper [33] applies the state-of-the-art 2D object detection on a color image first and uses 3D information to orient, place, and score bounding boxes around the objects. Amodal3Det [34] uses the color and depth images to extract features and build models to convert 2D results to 3D space. However, in practice, the rebuilt 3D models are always incomplete because of occlusion and reflection, which may lead to an inaccuracy in the regression. Our algorithm is inspired by the Amodal3Det method. To avoid the inaccuracy of an incomplete depth image, we combine this 2.5D fusion approach with the BEV image to express more 3D information.

## 3. Multi-Channel 3D Object Detection CNN

Since an image is a 3D projection in two dimensions, it is quite difficult to fully express 3D information. A depth image expresses the distance of an object to a camera plane, and a BEV image describes the distribution of the spatial object from a perspective perpendicular to the camera’s viewpoint. Therefore, the fusion of multiple kinds of information is beneficial for the better detection of objects in 3D space. In this paper, a 3D object detection CNN combining three-channel information is designed. RGB image, depth, and BEV images are used as the input of the network, and the object’s category, 3D size, and spatial position are regressed, respectively.

**Figure 1:**
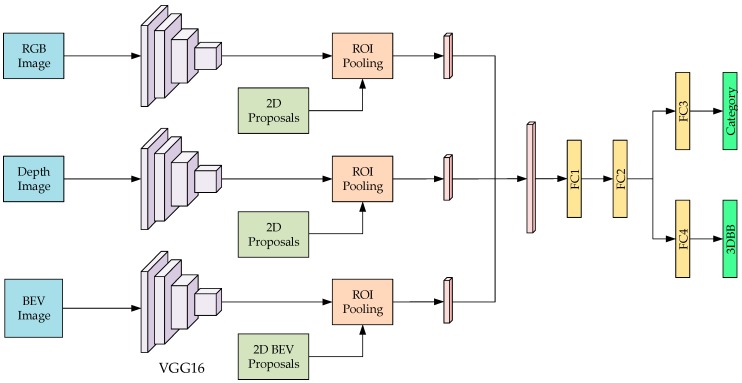
Multi-channel 3D object detection CNN architecture (ROI: region of interest, 3DBB: 3D bounding box).

The designed multi-channel object detection neural network system is shown in Figure 1. The Fast R-CNN is employed as the basic network structure to extend the 2D image object detection to the 3D object detection. The input is extended to three channels including an RGB image, a depth image, and a BEV image. VGG16 is utilized as the main convolutional network structure for feature extraction to enhance the learning of 3D spatial information. We employ the multiscale combinatorial grouping (MCG) [35] algorithm to generate many 2D proposals in the RGB image. As the depth image is the same view angle with the RGB image, it shares the same 2D proposals in the depth image. Then, we combine the 2D proposals with the depth information and semantic prior knowledge to generate 3D proposals, and then project them to the BEV plane. Finally, we can obtain 2D proposals for each channel. Then, the pre-obtained 2D proposals in each channel are utilized to generate the feature vectors with the same dimension using a single-layer spatial pyramid pooling layer, and then all vectors are connected as a whole vector. Finally, a multi-task regression is performed through two layers of fully connected layers to predict the object category and the 3D bounding box.

### 3.1. Input Data Generation for Convolutional Neural Network

In order to increase the network’s ability to perceive 3D information, a BEV image is generated from the RGB and depth images and it is used as an additional input to the convolutional neural network. Based on the processing methods of an RGB image as an input to a convolutional neural network, the depth and BEV images are quantized into the pixel range of [0,255], and then they are used as the input of the network.

The depth image acquired by the RGB-D sensor stores distance information, and the defined maximum depth is $z_{\max}$, and the depth value z was quantized to the image range $z^{\prime }$ as:

(1) $$z^{\prime }=\min(1,\frac{z}{z_{\max}})\cdot 255$$

**Figure 2:**
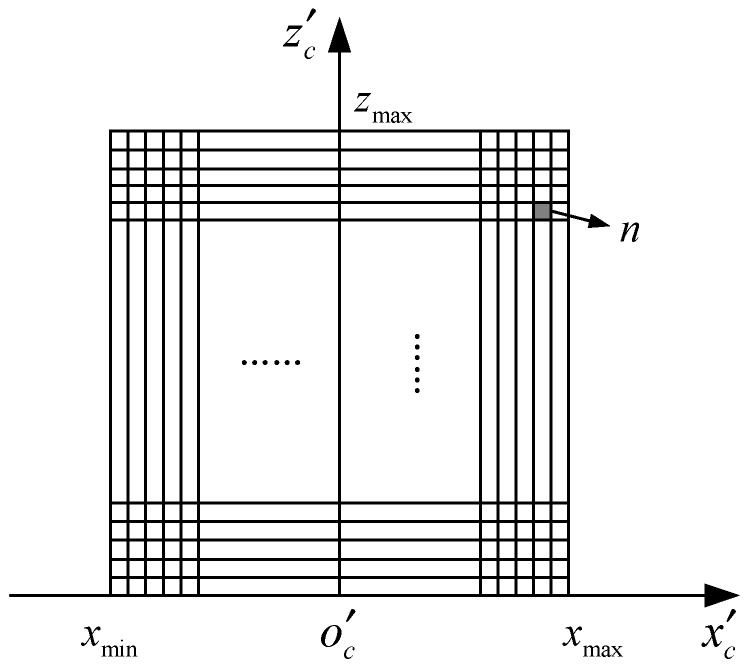
The coordinate system projected to the BEV image plane (a top-down view in the ground plane).

A point cloud is generated from the RGB and depth images, and it is projected onto the ground and rasterized to generate a BEV image. The BEV image is represented by the number of point clouds in each 2D grid cell. In a typical indoor environment, the range of depth images acquired by the RGB-D sensor is limited, so the range of the point cloud is limited to generate a BEV image of the same size. To illustrate it clearly, we provide the projected coordinate system of the camera to the BEV plane, as shown in Figure 2. The origin point ${o^{\prime }}_{c}$ is projected from the one of camera coordinate system and the orientations of ${o^{\prime }}_{c}{x^{\prime }}_{c}$ and ${o^{\prime }}_{c}{z^{\prime }}_{c}$ are oriented according to the ones of camera coordinate system. Let the coordinate system have a range of $\left[x_{\min},x_{\max}\right]$ in the *x*-axis direction, $\left[0,z_{\max}\right]$ in the *z*-axis direction, and δ is the resolution of the grid, then the size of the BEV image is $\left((x_{\max}-x_{\min})/\delta )\times (z_{\max}/\delta \right)$. Due to the different spatial point cloud density, the number of point clouds projected into each cell is very different, which is not conducive to data processing. Therefore, it is logarithmically transformed and converted to the image pixel range. Let the number of points in one cell of the BEV image be n and the maximum number of points in one cell be N, then the quantized image pixels are:

(2) $$p=\min(1,\frac{\mathrm{lg}(n+1)}{\mathrm{lg}N})\cdot 255/\delta$$

### 3.2. 2D and 3D Proposals Generation Based on Semantic Prior

The main idea of Fast R-CNN for object detection is to obtain the feature map of a whole image through the convolutional neural network and to employ the spatial pyramid pooling layer to extract features from the pre-generated 2D proposals in the image. The classifier is used to judge the features extracted in the proposal belonging to which category. The position of the proposal is adjusted by the regressor. This paper draws on the detection strategy based on the region proposal to extend 2D image detection to the spatial 3D object detection. Therefore, it is necessary to first acquire the 3D proposal of the object before performing category detection, 3D position, and size regression on this basis. In addition, it is also necessary to project the obtained 3D proposal back to BEV image to get a consistent proposal with the other input channels.

#### 3.2.1. 3D Proposal Parameter Representation

The regression of the 3D object is parameterized into a seven-dimensional vector $\left(x_{c},y_{c},z_{c},l,w,h,\theta \right)$. $\left(x_{c},y_{c},z_{c}\right)$ is the coordinate of the center point of the object bounding box in the camera coordinate system. (l,w,h) are the length, width, and height of the bounding box, respectively. θ is the angle (with the range of [−π/2,π/2]) between the *z*-axis direction of the camera coordinate system and the longer edge of the bounding box projected on the xz plane. The initial value of the object center point can be computed based on the point cloud corresponding to the proposal. Since noise always exists in measurement and data are often missed in the point cloud, the median value in the *z*-axis direction is taken as the initial value ${z^{\prime }}_{c}$, and ${x^{\prime }}_{c}$ and ${y^{\prime }}_{c}$ can be solved with the camera parameters:

(3) $$\{\begin{array}{c} {x^{\prime }}_{c}=\frac{\left(c_{x}-u\right)}{f_{x}}\cdot {z^{\prime }}_{c}\\ {y^{\prime }}_{c}=\frac{\left(y_{x}-u\right)}{f_{y}}\cdot {z^{\prime }}_{c} \end{array}$$

where $\left(c_{x},c_{y}\right)$ is the center point of the proposal in the image; (u,v) is the center point of the image; and $f_{x}$, $f_{y}$ are the focal lengths.

Since the point cloud of the proposal may contain a background point cloud other than the object, the error will be large if the initial size of the object is directly obtained by the point cloud. For common objects in the indoor environment, such as sofas and chairs, similar objects usually have similar dimensions; therefore, the average size of the objects on the dataset can be used as a priori knowledge to determine the size of the 3D proposal. In addition, since it is difficult to estimate the directional angle of the 3D proposal in the initial stage, all the initial angle values are set to zero for the sake of simplicity.

#### 3.2.2. Proposals Generation

The 3D object detection neural network proposed in this paper is based on the Fast R-CNN network structure. The single-layer spatial pyramid is utilized to pool the object proposal of different sizes in the feature map in order to obtain the output vector with the same dimension. Therefore, it is necessary to obtain object proposals in three channels separately. Since the RGB image and the depth image are acquired under the same view angle, the same proposal can be shared. However, the BEV image is obtained using the point cloud projection and has a constraint relationship with the RGB and depth images. Thus, it is vital to solve how to generate the object proposal in all three channels.

Searching for a suitable proposal box in a 3D space is usually computationally intensive and inefficient. Since the method of extracting the proposal from a 2D image is relatively mature, the 3D information can be acquired by combining the depth image. Multiscale combinatorial grouping [35] (MCG), which generates 2D bounding box proposals in RGB images, usually generates thousands of object proposals of different sizes. In the camera coordinate system, a depth image can be combined to generate a point cloud for each proposal box. According to the representation of the 3D proposal in Section 3.2.1, the center point of the proposal can be computed.

During training, the proposal samples needs to be divided into positive samples and negative samples. In order to determine the possible categories of proposals, the IoU (intersection over union) is calculated between the proposal and each 2D ground truth bounding box in the image, and the category corresponding to the largest value is selected. Inspired by the image semantic segmentation, we can obtain the pixel-level semantic category in the image and count the number of pixels with the same semantic category in the 2D proposal in order to analyze the probability for the proposal to belong to a certain category. This information can be used to judge the positive and negative samples when training. In order to better select the positive and negative samples, the ground truth in the RGB image is used to comprehensively calculate the category’s probability of each proposal. Let the number of pixels in the ground truth (belonging to the same category as the ground truth) bounding box be $n_{gt}$; the area of the bounding box be $S_{gt}$; and the number of pixels of the category in the intersection area be $n_{prop}$, which is the common area between the 2D proposal and the ground truth bounding box; and the proposal box area be $S_{prop}$ such that the score of the proposal belonging to the category can be calculated by the following formula:

(4) $$score=\frac{n_{prop}}{n_{gt}}\cdot \min(\frac{S_{prop}}{S_{gt}},\frac{S_{gt}}{S_{prop}})$$

The scores between the proposal box and each ground truth bounding box are calculated and the ground truth’s category corresponding to the maximum value is taken as the category of the proposal.

**Figure 3:**
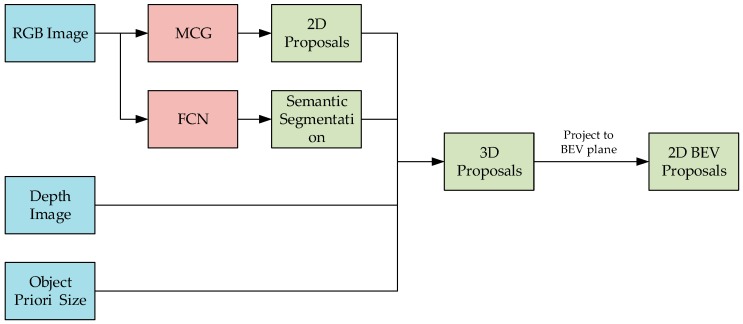
2D and 3D proposals generation.

A diagram of the system to generate the 2D and 3D proposals required for training is shown in Figure 3. First, the MCG algorithm is applied to obtain the 2D proposals in the image. The semantic segmentation of the image is obtained using the full convolutional neural network (FCN) algorithm. Then, the maximum possible category of the proposal is calculated. Finally, an initial value of the 3D proposal corresponding to each 2D proposal is obtained by combining the depth image and the a priori size of the object. In order to obtain the proposal in the BEV image, the 3D proposal is projected into the BEV image, and a BEV image proposal corresponding to each proposal in the image was obtained.

#### 3.2.3. 3D Bounding Box Regression of Objects

The designed multi-channel convolutional neural network extracts the features of the RGB image, depth image, and BEV image. The features in 2D proposals are transformed into uniform size vectors through the RoI pooling layer and connected to a whole vector. Finally, the predicted results of the network are obtained by the classifier and the 3D bounding box regressor. For each positive sample, the output of the network is relative to the ground truth bounding box, which is a seven-dimensional vector (Δx,Δy,Δz,Δl,Δw,Δh,Δθ). For each proposal, the ground truth category of the maximum probability is obtained according to the above method, and the normal value and the 3D proposal predicted value are applied for normalization:

(5) $$\{\begin{array}{c} \Delta x=\frac{x_{gt}-x_{c}}{l}\\ \Delta y=\frac{y_{gt}-y_{c}}{l}\\ \Delta z=\frac{z_{gt}-z_{c}}{l}\\ \Delta \theta =\frac{\theta_{gt}}{\pi }\cdot 180 \end{array}\{\begin{array}{c} \Delta l=\ln\frac{l_{gt}}{l}\\ \Delta w=\ln\frac{w_{gt}}{w}\\ \Delta h=\ln\frac{h_{gt}}{h} \end{array}$$

where $\left(x_{c},y_{c},z_{c},l,w,h,\theta \right)$ is the 3D proposal predicted value generated by the 2D proposal, and $\left(x_{gt},y_{gt},z_{gt},l_{gt},w_{gt},h_{gt},\theta_{gt}\right)$ is the ground truth of 3D bounding box corresponding to the maximum probability of the 2D proposal.

#### 3.2.4. Multi-Task Loss Function

For joint training classification and bounding box regression, the designed multitask loss function is:

(6) $$L=L_{cls}+\lambda L_{3DBB}$$

where $L_{cls}$ is the classification loss function, using the Softmax function. $L_{3DBB}$ is the 3D bounding box prediction-loss function, using the $L_{1}$ smooth-loss function. λ is the coefficient to balance the two loss function values.

## 4. Experiments

In order to verify the proposed multi-channel object detection neural network, the open source dataset NYU V2 is selected for experiments. The datasets are collected using an RGB-D camera in several indoor scenes. It consists of color and depth images and labeled 3D object bounding boxes. The training dataset contains 795 images and the test dataset contains 654 images. Zhuo et al. [34] re-label ground truths of the partial bounding boxes in some images in order to enhance the correct rate of the positive samples in images during training. Therefore, in order to facilitate fair experimental comparison with the work by Zhuo et al. [34], the modified NYUV2 dataset is adopted in the experiments.

### 4.1. Training Data Generation

To train the multi-channel neural network described in Section 3, the relevant training data needs to be prepared, such as RGB images, depth images, BEV images, 2D proposals in three channels, and the corresponding 3D proposals.

RGB and depth images are already included in the dataset. However, the BEV images need to be generated. As the NYUV2 dataset is collected by using a Kinect sensor, the range of the point cloud should be limited in order to acquire the same size for the BEV image obtained by the point cloud projection. In the camera coordinate system, the range of the *x*-axis direction is [−2.5 m,2.5 m], and the range of the *z*-axis direction is [0,5 m]. The point cloud is projected onto the plane to obtain a BEV image. The resolution of the grid is set as 0.01 m, then the size of the BEV image is 500 × 500 pixels.

In the RGB image, 2D proposals are generated by using the MCG algorithm. Since the depth image and the RGB image have the same viewpoint, the proposal of the depth image is consistent with the proposal of the image. The 3D proposal is generated according to the algorithm described in the third section, and the image semantic segmentation is done by using FCN. Then, the proposal is scored according to the ground truth of the 2D bounding box in the image and the proposal category is one of the bounding boxes with the highest score. A 3D proposal is generated based on the average size of each type of object on the training set as an initial value. The 3D proposal box is projected onto the BEV image to obtain a plan view proposal box corresponding to the 2D proposal frame in the image. Data augmentation is performed during training. The progress is that the 2D proposals are flipped horizontally in the image, and the corresponding proposals in the 3D proposals and the BEV image are simultaneously flipped.

Since the categories of semantic segmentation based on FCN in the NYUV2 dataset are not completely consistent with the categories of 3D object detection, FCN lacks two categories named "garbage bin" and "monitor" compared to the object categories in Zhuo et al.’s work [34]. Therefore, the two categories are removed and the remaining 17 categories were used.

### 4.2. Training Parameter Setting

The Caffe framework is applied during training, and the pre-trained VGG16 model on ImageNet is utilized to initialize the parameters of the forward channel of the network. The coefficient *λ* in the loss function is set to 1; the base learning rate is set to 0.0005; and the learning strategy is "step", which is multiplied by 0.1 every 10,000 iterations. The stochastic gradient descent algorithm is employed to implement 40,000 iterations. Two images are randomly selected in each batch, 128 proposals are randomly selected in each image, and the ratio of positive samples to negative samples is 1:3. It takes about 4 h to iterate 40,000 times in an NVIDIA DGX Station (Tesla V100 GPU, NVIDIA, Santa Clara, CA, USA). During the test, the forward channel average inference time is around 0.18 s.

### 4.3. Experimental Results and Analysis

**Figure 4:**
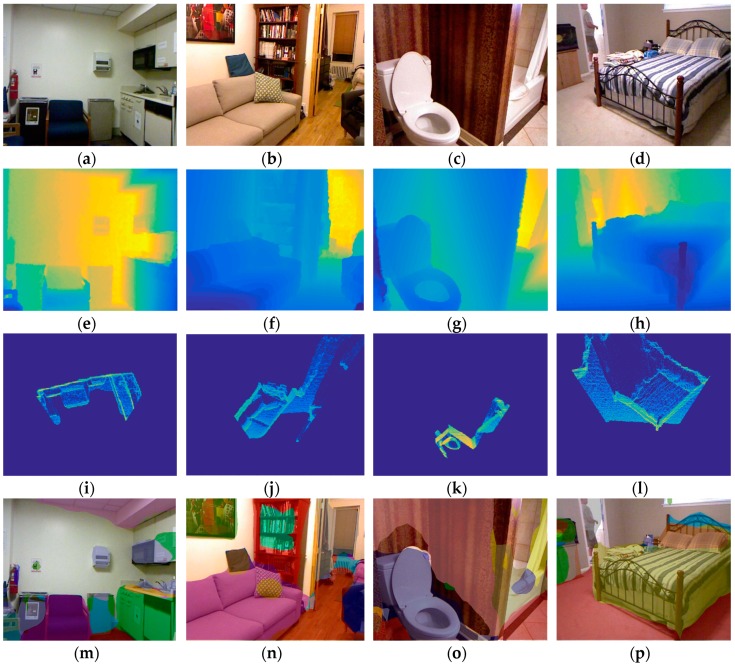

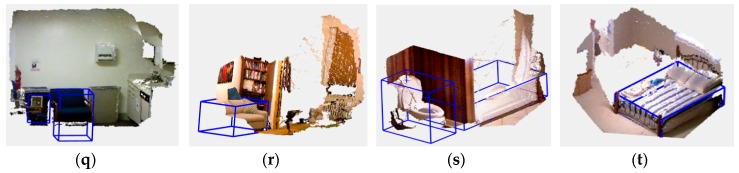
Experiments on the modified NYUV2 dataset where four different categories (chair, sofa, toilet, and bed) are detected. The first row (**a**–**d**) shows RGB images, the second row (**e**–**h**) shows depth images, the third row (**i**–**l**) shows BEV images, the fourth row (**m**–**p**) shows semantic images, and the last row (**q**–**t**) shows the point cloud with detected 3D bounding boxes.

Experiments are carried out on the modified NYUV2 dataset [34] and they are compared with two recently related papers [27,34]. The experimental results are shown in Table 1. The proposed algorithm improves the average accuracy compared with the previous works [27,34], and the performance of the algorithm after adding the BEV image is also verified. In most categories, the accuracy is improved by several percent, especially for objects that are relatively independent in the BEV image, such as beds, chairs, and sofas. To illustrate the details of the algorithm, we conduct an ablation study on the dataset. The results are shown in Table 2. We remove the BEV channel to verify the effect. Furthermore, we can observe that the average accuracy is improved by 3.1% with the BEV channel. To observe the results more intuitively, we give some examples with all the input images and results, as shown in Figure 4. It shows the detection results in four different categories (chair, sofa, toilet, and bed). The figure shows the input three channel images (RGB, depth, and BEV images), semantic segmentation images, and point cloud images in which the detected 3D object bounding boxes are displayed. From the detected 3D bounding boxes, we can observe that the sizes and positions of different objects are estimated well. 

To verify the performance of the algorithm sufficiently, we carry out another experiment on the SUN RGB-D [18] dataset, which contains 10,335 RGB-D images and 64,595 3D bounding boxes. We train the model using our method and compares it with other previous works [27,36], which are also tested in the same dataset. The experimental results are shown in Table 3 and it can be observed that our method can achieve better performance in most categories and had a certain improvement in the mean average precision.

**Figure 5:**
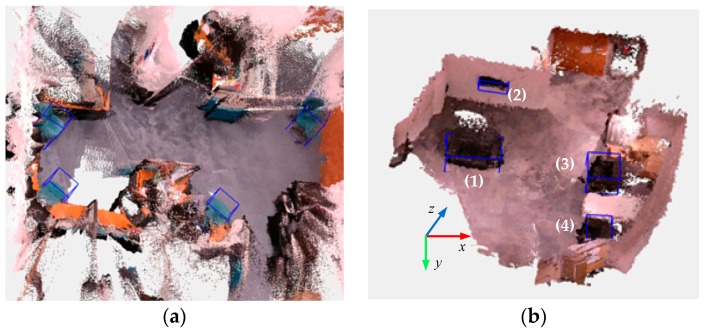

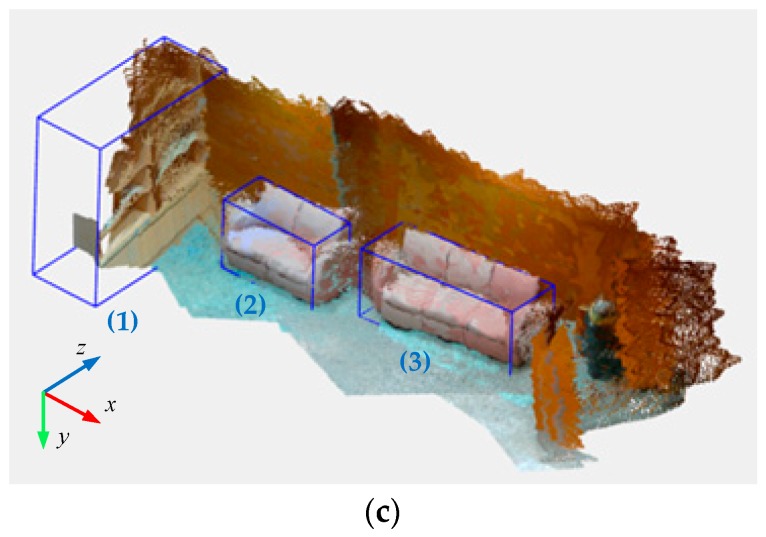
Experiments in a service robot in three indoor scenes: (**a**) an office room, where four chairs are detected; (**b**) an office corner, where three sofas and a television are detected; and (**c**) another corner in an office, where a bookshelf and two sofas are detected. The coordinate systems in (**b**) and (**c**) were the global systems.

In order to utilize the 3D object detection for indoor robot environmental perception, we conduct experiments in a service robot in three indoor scenes. A Kinect RGB-D sensor is used in the robot to collect RGB and depth images. As the robot moves in the environment, the ORB-SLAM2 [37,38] algorithm with a dense point cloud is employed to estimate poses of the robot and establish a dense map in our experiments. Then, the 3D object detection algorithm in this paper is implemented only in the keyframes of ORB-SLAM to decrease the computation. Because one object can be observed in several keyframes, the 3D bounding boxes of objects are transformed into the system coordinates and the average position and size of the same object’s 3D bounding boxes are calculated. The experimental results are shown in Figure 5. 3D maps are constructed and several 3D object bounding boxes are shown in each Figure. In Figure 5a, four chairs are detected in an office; in Figure 5b, three sofas and a television are detected; and in Figure 5c, a bookshelf and two sofas are detected. In order to measure the results of the algorithm quantitatively, the estimated 3D bounding boxes of objects are given and compared with the ground truth, as shown in Table 4. To facilitate measurement, we select Figure 5b,c to conduct the test as the 3D bounding boxes of objects in these scenes are almost parallel to each other and it is easy to measure the ground truth. The Kinect RGB-D sensor is fixed on the robot with the height of 1.2 m. We keep the sensor parallel to the object at the beginning when conducting the ORB-SLAM2 algorithm and set the coordinate system of the first keyframe as the global one (the coordinate system drawn in Figure 5b,c). Then, we transfer the estimated results in each keyframe to the global coordinate system. In Table 4, we give the 3D bounding box ground truth of objects and the estimated results using our method in the global coordinate system. Finally, we calculate the 3D IoU (intersection over union) of 3D bounding boxes to measure the accuracy. The average 3D IoU is 0.61 and we can observe that our method detect the sofa with higher accuracy than the bookshelf and television. During the 3D object detection, it takes about 2.2 s to regress the 3D bounding box with a Tesla V100 GPU (about 1.6 s to extract region proposals in each keyframe based on MCG, 0.42 s to obtain the semantic segmentation using FCN, and 0.18 s to run the forward inference). Experimental results verify that the proposed algorithm can benefit robot environmental perception using 3D object detection.

## 5. Conclusions

Aiming at the problem of 3D object detection for an indoor service robot’s environmental perception, this paper proposes a multi-channel convolutional neural network that combines the RGB, depth, and BEV images together. By adding the BEV image channel, the perception ability of the neural network was improved. Training and testing are done on the modified NYUV2 dataset and SUN RGB-D dataset. Based on the experimental results, our algorithm is superior compared with the two recent papers. Furthermore, the actual experiments in the service robot also demonstrate that the proposed 3D object detection algorithm had the potential to be applied in indoor service robots for better environmental perception. As it has a process of 2D proposals generation in the method, the future work will focus on the end-to-end method to achieve the 3D object detection using a CNN.

## Figures and Tables

**Table 1:** 3D object detection comparisons on the modified NYUV2 dataset (mAP: mean average precision).

Methods	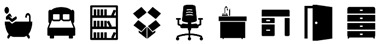									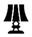	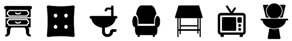							mAP
[27]	62.3	81.2	23.9	3.8	58.2	24.5	36.1	0.0	31.6	28.7	54.5	38.5	40.5	55.2	43.7	1.0	76.3	38.8
[34]	36.1	84.5	40.6	4.9	46.4	44.8	33.1	10.2	44.9	29.4	60.6	46.3	58.3	61.8	43.2	16.3	79.7	43.6
Ours	36.6	88.4	41.5	6.4	55.3	46.7	37.2	8.2	43.4	35.9	62.1	49.7	61.4	65.3	47.8	20.4	83.8	46.5

**Table 2:** An ablation study on the modified NYUV2 dataset (mAP: mean average precision).

Methods	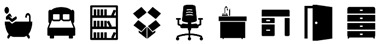									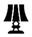	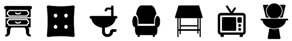							mAP
Img+Depth	35.8	84.2	40.5	4.7	46.9	43.4	33.0	10.5	44.6	28.8	61.1	45.9	58.5	61.7	43.6	15.5	78.3	43.4
Img+Depth+BEV	36.6	88.4	41.5	6.4	55.3	46.7	37.2	8.2	43.4	35.9	62.1	49.7	61.4	65.3	47.8	20.4	83.8	46.5

**Table 3:** 3D object detection comparisons on the SUN RGB-D dataset (mAP: mean average precision).

Methods	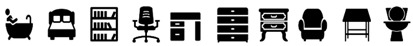										mAP
[27]	44.2	78.8	11.9	61.2	20.5	6.4	15.4	53.5	50.3	78.9	42.1
[36]	58.3	63.7	31.8	62.2	45.2	15.5	27.4	51.0	51.3	70.1	47.6
Ours	55.4	73.6	26.2	64.3	43.1	17.3	24.5	54.9	53.7	75.4	48.8

**Table 4:** Quantitative experiments of 3D object detection in indoor scenes ^1^.

Figure 5		3D Bounding Box (Ground Truth)							3D Bounding Box (Estimation)							3D IoU
		*x_gt_*	*y_gt_*	*z_gt_*	*l_gt_*	*w_gt_*	*h_gt_*	*θ_gt_*	*x*	*y*	*z*	*l*	*w*	*h*	*θ*	
(b)	(1)	1.20	−0.85	2.36	1.44	0.72	0.70	0	1.24	−0.87	2.28	1.51	0.80	0.75	7.6	0.70
	(2)	1.50	0.20	4.25	0.95	0.15	0.55	0	1.41	0.18	4.17	0.88	0.22	0.61	5.9	0.33
	(3)	4.20	−0.85	2.10	0.85	0.68	0.70	−90	4.17	−0.91	2.17	0.91	0.77	0.76	−75.4	0.63
	(4)	4.20	−0.85	0.20	0.85	0.68	0.70	−90	4.28	−0.80	0.27	0.71	0.62	0.57	−83.4	0.54
(c)	(1)	−1.02	−0.20	2.00	2.00	0.41	2.10	90	−0.88	−0.24	1.91	2.11	0.48	2.02	83.1	0.46
	(2)	0.75	−0.80	2.30	1.35	0.75	0.80	0	0.71	−0.82	2.27	1.40	0.80	0.77	1.8	0.83
	(3)	3.00	−0.80	2.50	1.92	0.75	0.80	0	2.94	−0.83	2.52	1.95	0.80	0.87	2.7	0.80
Average 3D IoU																0.61

^1^ The units of *θ_gt_* and *θ* are °, and the others are m.

## References

[1] Krizhevsky A., Sutskever I., Hinton G. Imagenet classification with deep convolutional neural networks. Proceedings of the Conference on Neural Information Processing Systems (NIPS 2012).

[2] Szegedy C., Liu W., Jia Y., Sermanet P., Reed S., Anguelov D., Erhan D., Vanhoucke V., Rabinovich A. Going deeper with convolutions. Proceedings of the 2015 IEEE Conference on Computer Vision and Pattern Recognition (CVPR 2015).

[3] He K., Zhang X., Ren S., Sun J. Deep residual learning for image recognition. Proceedings of the 2016 IEEE Conference on Computer Vision and Pattern Recognition (CVPR 2016).

[4] Girshick R., Donahue J., Darrell T., Malik J. Rich feature hierarchies for accurate object detection and semantic segmentation. Proceedings of the 2014 IEEE Conference on Computer Vision and Pattern Recognition (CVPR 2014).

[5] Girshick R. Fast R-CNN. Proceedings of the 2015 IEEE International Conference on Computer Vision (ICCV 2015).

[6] Ren S., He K., Girshick R., Sun J. (2017). Faster R-CNN: Towards real-time object detection with region proposal networks. IEEE Trans. Pattern Anal. Mach. Intell..

[7] He K., Zhang X., Ren S., Sun J. (2015). Spatial pyramid pooling in deep convolutional networks for visual recognition. IEEE Trans. Pattern Anal. Mach. Intell..

[8] Geiger A., Lenz P., Urtasun R. Are we ready for autonomous driving? The KITTI vision benchmark suite. Proceedings of the 2012 IEEE Conference on Computer Vision and Pattern Recognition (CVPR 2012).

[9] Geiger A., Lenz P., Stiller C., Urtasun R. (2013). Vision meets robotics: The KITTI dataset. Int. J. Robot. Res..

[10] Qi C.R., Liu W., Wu C., Su H., Guibas L.J. Frustum PointNets for 3D object detection from RGB-D Data. Proceedings of the 2018 IEEE Conference on Computer Vision and Pattern Recognition (CVPR 2018).

[11] Zhou Y., Tuzel O. VoxelNet: End-to-end learning for point cloud based 3D object detection. Proceedings of the 2018 IEEE Conference on Computer Vision and Pattern Recognition (CVPR 2018).

[12] Xiang Y., Choi W., Lin Y., Savarese S. Data-driven 3D voxel patterns for object category recognition. Proceedings of the 2015 IEEE Conference on Computer Vision and Pattern Recognition (CVPR 2015).

[13] Chen X., Kundu K., Zhang Z., Ma H., Fidler S., Urtasun R. Monocular 3D object detection for autonomous driving. Proceedings of the 2016 IEEE Conference on Computer Vision and Pattern Recognition (CVPR 2016).

[14] Chen X., Zhu Y. 3D object proposals for accurate object class detection. Proceedings of the Conference on Neural Information Processing Systems (NIPS 2015).

[15] Chen X., Ma H., Wan J., Li B., Xia T. Multi-view 3D object detection network for autonomous driving. Proceedings of the 2017 IEEE Conference on Computer Vision and Pattern Recognition (CVPR 2017).

[16] Ku J., Mozifian M., Lee J., Harakeh A., Waslander S.L. Joint 3D proposal generation and object detection from view aggregation. Proceedings of the 2018 IEEE/RSJ International Conference on Intelligent Robots and Systems (IROS 2018).

[17] Silberman N., Hoiem D., Kohli P., Fergus R. Indoor segmentation and support inference from RGBD images. Proceedings of the 12th European Conference on Computer Vision (ECCV 2012).

[18] Song S., Lichtenberg S.P., Xiao J. SUN RGB-D: A RGB-D scene understanding benchmark suite. Proceedings of the 2015 IEEE Conference on Computer Vision and Pattern Recognition (CVPR 2015).

[19] Zhuang Y., Lin X.Q., Hu H.S., Guo G. (2015). Using scale coordination and semantic information for robust 3-D object recognition by a service robot. IEEE Sens. J..

[20] Lin C.-M., Tsai C.-Y., Lai Y.-C., Li S.-A., Wong C.-C. (2018). Visual object recognition and pose estimation based on a deep semantic segmentation network. IEEE Sens. J..

[21] Felzenszwalb P.F., Girshick R.B., McAllester D., Ramanan D. (2010). Object detection with discriminatively trained part-based models. IEEE Trans. Pattern Anal. Mach. Intell..

[22] Redmon J., Divvala S., Girshick R., Farhadi A. You only look once: Unified, real-time object detection. Proceedings of the 2016 IEEE Conference on Computer Vision and Pattern Recognition (CVPR 2016).

[23] Redmon J., Farhadi A. YOLO9000: Better, Faster, Stronger. Proceedings of the 2017 IEEE Conference on Computer Vision and Pattern Recognition (CVPR 2017).

[24] Redmon J., Farhadi A. (2018). YOLOv3: An incremental improvement. arXiv.

[25] Dai J., Li Y., He K., Sun J. R-FCN: Object detection via region-based fully convolutional networks. Proceedings of the Conference on Neural Information Processing Systems (NIPS 2016).

[26] Engelcke M., Rao D., Wang D., Tong C., Posner I. Vote3Deep: Fast object detection in 3D point clouds using efficient convolutional neural networks. Proceedings of the 2017 IEEE International Conference on Robotics and Automation (ICRA 2017).

[27] Song S., Xiao J. Deep sliding shapes for amodal 3D object detection in RGB-D images. Proceedings of the 2016 IEEE Conference on Computer Vision and Pattern Recognition (CVPR 2016).

[28] Qi C.R., Su H., Mo K., Guibas L.J. PointNet: Deep learning on point sets for 3D classification and segmentation. Proceedings of the 2017 IEEE Conference on Computer Vision and Pattern Recognition (CVPR 2017).

[29] Qi C.R., Yi L., Su H., Guibas L.J. PointNet++: Deep hierarchical feature learning on point sets in a metric space. Proceedings of the Conference on Neural Information Processing Systems (NIPS 2017).

[30] Qi C.R., Su H., Nießner M., Dai A., Yan M., Guibas L.J. Volumetric and multi-view CNNs for object classification on 3D data. Proceedings of the 2016 IEEE Conference on Computer Vision and Pattern Recognition (CVPR 2016).

[31] Enzweiler M., Gavrila D.M. (2011). A multi-level mixture-of-experts framework for pedestrian classification. IEEE Trans. Image Process..

[32] Gonzalez A., Vazquez D., Lopez A.M., Amores J. (2017). On-board object detection: Multicue, multimodal, and multiview random forest of local experts. IEEE Trans. Cybern..

[33] Lahoud J., Ghanem B. 2D-driven 3D object detection in RGB-D images. Proceedings of the 2017 IEEE International Conference on Computer Vision (ICCV 2017).

[34] Zhuo D., Latecki L.J. Amodal detection of 3D objects: Inferring 3D bounding boxes from 2D ones in RGB-Depth images. Proceedings of the 2017 IEEE Conference on Computer Vision and Pattern Recognition (CVPR 2017).

[35] Arbelaez P., Pont-Tuset J., Barron J., Marques F., Malik J. Multiscale combinatorial grouping. Proceedings of the 2014 IEEE Conference on Computer Vision and Pattern Recognition (CVPR 2014).

[36] Ren Z., Sudderth E.B. Three-dimensional object detection and layout prediction using clouds of oriented gradients. Proceedings of the 2016 IEEE Conference on Computer Vision and Pattern Recognition (CVPR 2016).

[37] Mur-Artal R., Montiel J.M.M., Tardos J.D. (2015). ORB-SLAM: A versatile and accurate monocular SLAM system. IEEE Trans. Rob..

[38] Mur-Artal R., Tardos J.D. (2017). ORB-SLAM2: An open-source SLAM system for monocular, stereo, and RGB-D cameras. IEEE Trans. Rob..

